# A Case of Ramucirumab for Radiation Necrosis Following Stereotactic Radiotherapy for Brain Metastases From Lung Cancer

**DOI:** 10.7759/cureus.70460

**Published:** 2024-09-29

**Authors:** Tatsuya Takeda, Daisuke Nakamura, Takaya Ikeda

**Affiliations:** 1 Department of Radiology, National Hospital Organization (NHO) Nagasaki Medical Center, Nagasaki, JPN; 2 Department of Radiology, Nagasaki University Hospital, Nagasaki, JPN; 3 Department of Respiratory Medicine, National Hospital Organization (NHO) Nagasaki Medical Center, Nagasaki, JPN

**Keywords:** brain metastasis, brain radiation necrosis, ramucirumab, stereotactic radiotherapy (srt), vegf

## Abstract

It is well known that bevacizumab is effective against radiation necrosis in the brain (hereafter referred to as brain necrosis). Herein, we report a case of brain necrosis in a patient treated with a regimen that included ramucirumab, an anti-vascular endothelial growth factor (VEGF) inhibitor. A woman in her 40s presented with five brain metastases from lung adenocarcinoma at the initial diagnosis. Each metastasis was treated with stereotactic radiotherapy. Subsequent magnetic resonance imaging showed increased oedema surrounding the lesion in the left frontal lobe, leading to a diagnosis of radiation-induced brain necrosis. Docetaxel + ramucirumab was chosen for third-line chemotherapy. During treatment, perioperative brain necrotic oedema diminished. Furthermore, anti-VEGF inhibitor regimens should be considered for reducing oedema associated with radiation necrosis of the brain, as they can be utilized alongside chemotherapy for the primary tumor.

## Introduction

Brain metastases are reported to occur in approximately 10% of patients with cancer, with lung cancer brain metastases accounting for 40-50% of all brain metastases [1]. Radiotherapy plays an important role in the treatment of brain metastases, and the local control rate has increased due to the widespread use of stereotactic radiotherapy (SRT) [2]. Moreover, radiation brain necrosis, an adverse event, occurs in 7-24% of patients due to an increased local dose [3,4]. Steroids can provide some benefits for cerebral oedema, but dose reduction can cause symptom flare-ups [5,6]. Notably, bevacizumab, an anti-vascular endothelial growth factor (VEGF) inhibitor, has been reported to be useful [7].

Here, we report a case of radiation necrosis of the brain after stereotactic radiotherapy for brain metastases from lung cancer, in which ramucirumab, an anti-VEGF inhibitor, was effective.

## Case presentation

**Figure 1:**
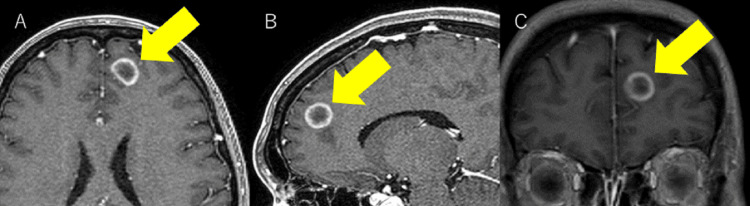
Left anterior frontal lesion before radiotherapy (A) axial, (B) sagittal, (C) coronal

A 45-year-old female presented at her local physician's office with symptoms of nasal discharge and a cough. As there was no improvement, she revisited the hospital, where chest radiography revealed a mass shadow and significant pleural effusion extending from the right hilum to the mediastinum. Subsequently, she was referred to the Department of Pulmonary Medicine at our hospital. Here, computed tomography (CT) identified a mass in the right upper lobe and right hilum, accompanied by pleural thickening and effusion, indicative of cancerous pleuritis. The diagnosis of adenocarcinoma (epidermal growth factor receptor (EGFR) mutation exon 19 deletion, programmed death ligand 1 (PD-L1) <1%, c-ros oncogene (ROS1) negative, anaplastic lymphoma kinase (ALK) negative) was established through pleural fluid cytology and bronchoscopy. Additionally, contrast-enhanced magnetic resonance imaging (MRI) of the head showed multiple brain metastases (Figure 1), leading to a diagnosis of cT4N2M1c stage IVB (Union for International Cancer Control (UICC) 8).

**Figure 2:**
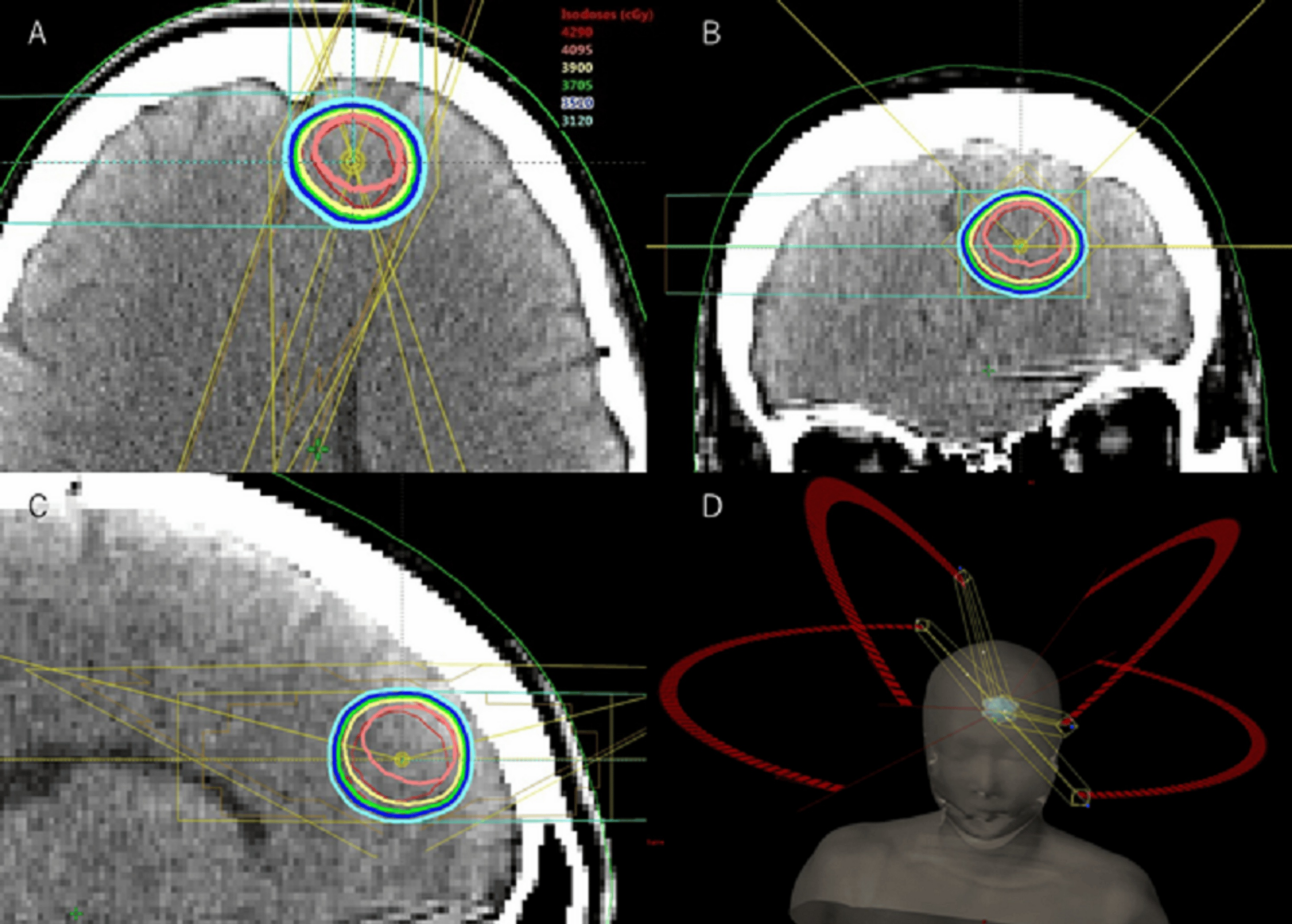
Stereotactic radiotherapy planning Stereotactic radiotherapy (SRT) 39Gy/13fr. at dynamic conformal arc (85% isodose cover) (A) axial, (B) coronal, (C) sagittal, (D) overview

The first-line treatment consisted of osimertinib from November 2019 to December 2020, followed by cisplatin plus pemetrexed as second-line therapy from December 2020 to March 2021. In November 2019, SRT was administered for brain metastases using a dynamic conformal arc technique (SRT, 39 Gy/13 fr. to the anterior left frontal lobe with 85% isodose coverage) (Figure 2).

**Figure 3:**
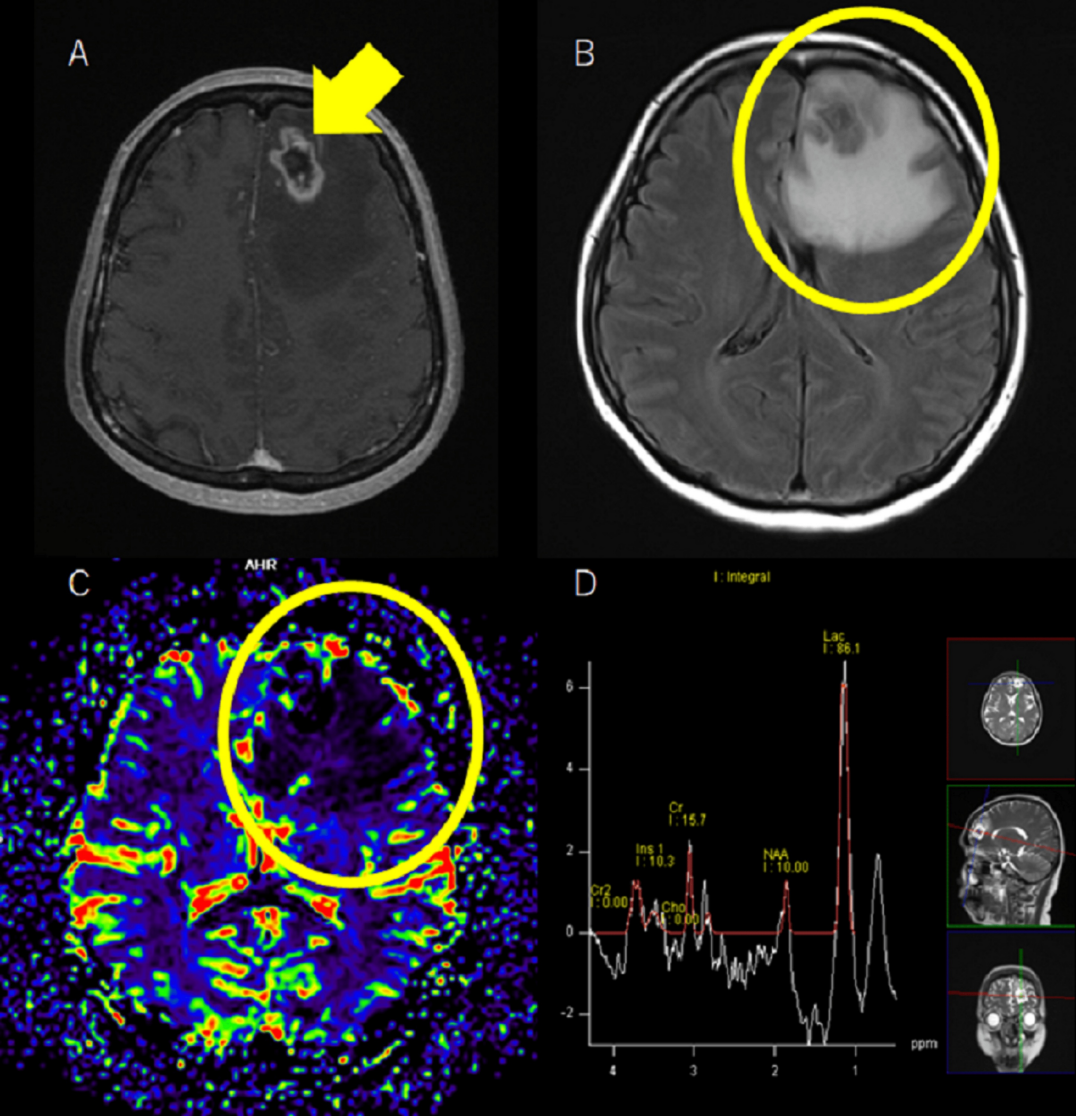
Left anterior frontal lesion before radiotherapy (A) gadolinium-enhanced T1-weighted imaging (Gd-T1WI), (B) fluid-attenuated inversion recovery (FLAIR), (C) magnetic resonance (MR) perfusion, (D) magnetic resonance spectroscopy (MRS)

SRT was also applied to three additional sites, which are not detailed here. The lesion in the left frontal lobe was reducing in size until April 2020; however, it enlarged and exhibited increased periapical oedema from June onwards. The volume of the contrast-enhanced T1-weighted imaging (T1WI) lesion exceeded that of the fluid-attenuated inversion recovery (FLAIR) lesion in imaging assessments, with oedema volume (135843 mm3) over contrast-enhanced T1WI lesion volume (7507 mm3) resulting in a quotient of 18.1, which is greater than 10 [8]. The lesion quotient, calculated as T2-weighted imaging (T2WI) nodal lesion area (96.5 mm2) over contrast-enhanced T1WI lesion area (438.6 mm2)= 0.22≤0.3 [9]. Magnetic resonance (MR) perfusion showed decreased cerebral blood volume and magnetic resonance spectroscopy (MRS) showed lactate (Lac)/choline (Cho)=33.1 >1.05 (Figure 3) [10].

**Figure 4:**
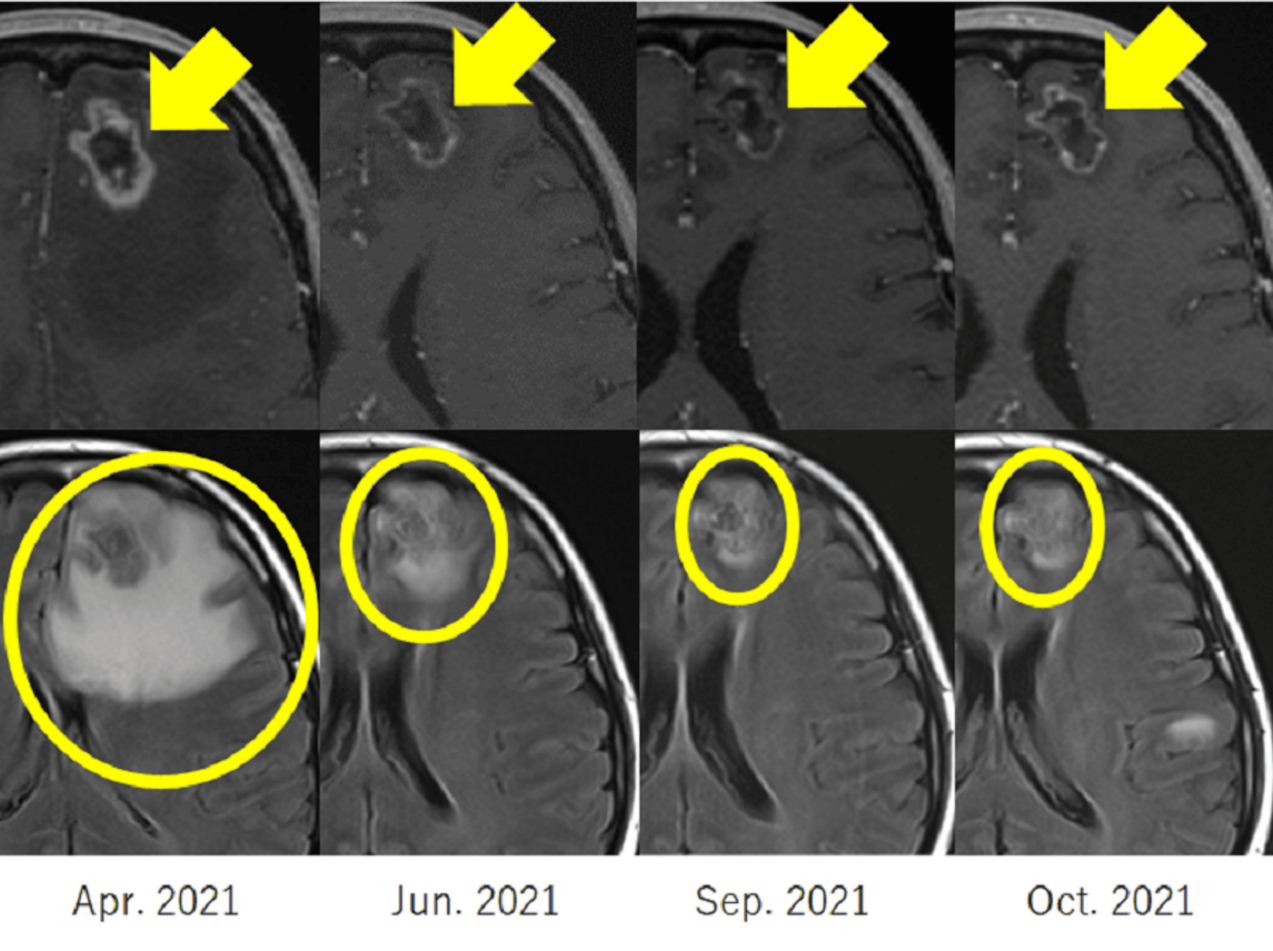
Course of brain necrosis upper: gadolinium-enhanced T1-weighted imaging (Gd-T1WI), lower: fluid-attenuated inversion recovery (FLAIR)

**Figure 5:**
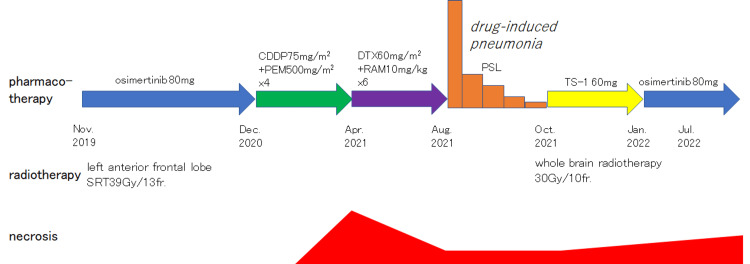
Course of treatment CDDP: cisplatin, PEM: pemetrexed, DTX: docetaxel, RAM: ramucirumab, PSL: prednisolone, SRT: stereotactic radiotherapy

Based on these findings and other observations, the final diagnosis was radiation-induced brain necrosis. As no symptoms were observed, anti-oedema medications were not administered. Concurrently, the patient’s second-line therapy proved ineffective; thus, a third-line treatment was selected. Given the therapeutic mechanism of ramucirumab, treatment with docetaxel plus ramucirumab was initiated in April 2021 to potentially mitigate the anti-oedema effects associated with radiation brain necrosis. Subsequent MRI findings in the following month indicated a trend toward reduction in the lesion and surrounding oedema. Treatment continued until July 2021 but was halted in August due to drug-induced pneumonia, necessitating the initiation of steroids. After resolving the drug-induced pneumonia by October, the patient transitioned to TS-1 as the fourth-line treatment. She also underwent whole-brain irradiation at 30 Gy/10 fractions in October 2021 for multiple brain metastases. The patient was transitioned to osimertinib in January 2022. Despite the emergence of additional brain metastases, the oedema around the brain necrosis only increased slightly (Figure 4), and no symptoms related to the brain necrosis manifested until her passing in December 2022 (Figure 5).

## Discussion

Because radiotherapy often serves as the primary treatment for brain metastases, radiation-induced brain necrosis, which occurs at a relatively high rate, represents an adverse event that must not be overlooked. Notably, this complication is expected to become more prevalent as patient survival times are often extended due to advancements in chemotherapy and other factors. Specifically, tyrosine kinase inhibitors have been suggested to increase the risk of radiation brain necrosis, and it is essential to be cognizant of this increased risk, given the growing number of indications in lung cancer [11].

There are currently no established treatments for radiation-induced brain necrosis. Conventionally, drug therapies, primarily steroid hormones, are administered first [12]. Surgical resection is recommended when the patient’s condition does not improve or is ineffective [13]. For example, steroids are prone to adverse effects, such as long-term use and relapse after discontinuation. Conversely, surgical resection is fraught with problems such as the risk of further deterioration of the patient's neurological condition as a result of surgery and the question of whether surgery should be performed in many patients with a poor prognosis. In this context, the report that the anti-VEGF inhibitor, bevacizumab, is effective against radiation-induced brain necrosis has led to a wide range of options that do not involve surgical resection. Irradiation-induced necrosis has also been reported to be caused by periastrocytes expressing VEGF [14], with further evidence for its effect on bevacizumab [15]. A similar mechanism of action is expected for ramucirumab, which has similar efficacy.

Although treating brain necrosis following brain metastases treatment from lung cancer is challenging, aflibercept may be utilized for various forms of brain necrosis. This medication, previously employed for ocular oedema, including macular oedema, is anticipated to be effective against brain oedema in brain necrosis. VEGF receptor tyrosine kinase inhibitors, such as axitinib and sunitinib, are also effective in managing brain oedema associated with brain necrosis [16]; however, they do not prevent brain necrosis [17]. Furthermore, it is advisable to consider VEGF-related molecularly targeted drugs for brain necrosis across a spectrum of primary diseases.

Distinguishing brain necrosis from the recurrence or enlargement of brain metastases presents a challenge. It is established that the gold standard for diagnosis is pathological evaluation from surgical specimens; nevertheless, the reasons mentioned previously often preclude surgery. Consequently, clinical observations and MRI assessments, including contrast-enhanced MRI, serve as the primary diagnostic tools, with several indicators currently employed. Despite these methods, definitive diagnosis remains elusive in many cases. To address this, research is underway to distinguish cases using nuclear medicine techniques. Various positron emission tomography (PET) tests, such as [18F]fluorodeoxyglucose (FDG), [11C]methionine (MET), [18F]fluoroethyl-l-tyrosine (FET), [11C]choline, [18F]dihydroxyphenylalanine (DOPA), [18F]fluorothymidine (FLT), and [201Tl]single-photon emission computed tomography (SPECT), have been documented, with existing reviews and analyses on these tests [18-20]. Ideally, these nuclear medicine tests should be used in combination when feasible. Amino acid PET is considered particularly valuable for differential diagnosis, yet its clinical application is limited by the availability of facilities. Currently, only [201Tl]SPECT and [18F]FDG PET are practicable, with their low sensitivity and specificity noted. It is hoped that the use of amino acid PET in clinical settings will expand and become routine.

## Conclusions

We encountered a case where ramucirumab was effective in treating radiation necrosis of the brain. Its efficacy was also targeted in combination with chemotherapy for the primary tumor. Therefore, we believe that a regimen including an anti-VEGF inhibitor should be considered for patients with brain radiation necrosis if this is an option.
